# 中国肺癌低剂量CT筛查指南（2025年版）

**DOI:** 10.3779/j.issn.1009-3419.2025.102.43

**Published:** 2025-12-20

**Authors:** 中国肺癌早诊早治专家组 中国西部肺癌研究协作中心

**Keywords:** 肺肿瘤, 筛查, 低剂量计算机断层扫描, 指南, 中国, Lung neoplasms, Screening, Low-dose computed tomography, Guideline, China

## Abstract

肺癌是导致中国癌症死亡的首要原因。通过优化肺癌筛查策略，提高筛查效率，减少相关危害已成为当前肺癌筛查的研究热点和方向。本文的目的是对2023年中国肺癌低剂量计算机断层扫描（low-dose computed tomography, LDCT）筛查指南进行修订。由国家卫健委指定的中国肺癌早诊早治专家组专家及部分中国西部肺癌研究协作中心专家，共同参与了2025年版中国肺癌LDCT筛查指南的修订工作。专家组根据近年来国内外LDCT肺癌筛 查进展，结合我国肺癌流行病学特征，共同修订了本肺癌筛查指南。本指南对以下内容进行了修订：（1）高危人 群定义中明确了职业暴露年限，另外，如果在年度筛查中发现受检者身体状况无法耐受可能的肺癌切除手术，或罹患其他严重威胁生命的疾病，则建议停止LDCT筛查；（2）更新了结节大小的测量方法，将平均直径作为结节大小的衡量指标；（3）结节管理中，将基线筛查中实性结节或部分实性结节实性成分的平均直径阳性阈值提高至6 mm，并将实性结节或部分实性的阳性结节复查时间由6个月调整为3-6个月；（4）建议将知情同意和共同决策贯穿于高危人群选择、筛查间隔和结节管理环节。本次修订进一步明确和优化了高危人群选择及结节管理，并在对肺癌筛查获益和危害充分权衡基础上，强调了知情同意和共同决策的重要性。本次修订将使得LDCT筛查指南更适应我国国情，并提升了我国肺癌筛查的规范性与适用性。

## Abstract (en)

Lung cancer remains the leading cause of cancer-related deaths in China. Optimizing lung cancer screening strategies to improve screening efficiency and mitigate associated harms has become a key research focus and priority in current clinical practice. The purpose of this paper is to revise the China national lung cancer screening guideline with low-dose computed tomography (LDCT) (2023 version). The revision work for the 2025 version of this guideline was jointly undertaken by experts from the Chinese Expert Group on Early Diagnosis and Treatment of Lung Cancer and experts from the China Lung Oncology Group. Drawing on recent advances in LDCT lung cancer screening at home and abroad, combined with the epidemiological characteristics of lung cancer in China, the expert panel revised the guideline with the following key updates: (1) In the selection criteria of high-risk population, the exposure duration to occupational lung carcinogens is clearly specified; additionally, if an individual’s physical condition cannot tolerate potential lung cancer resection surgery or the individual suffers from a life-threatening disease during annual screening, discontinuation of LDCT screening is recommended. (2) The method for measuring nodule size is updated, with mean diameter adopted as the metric for assessing nodule size. (3) In nodule management, the positive threshold for the mean diameter of solid nodules or the solid components of part-solid nodules in baseline screening is raised to 6 mm, and the follow-up interval for positive solid or part-solid nodules is adjusted from 6 months to 3-6 months. (4) It is recommended that informed consent and shared decision-making be integrated throughout the entire process, including high-risk group identification, screening interval determination, and nodule management. This revision further clarifies and optimizes the selection of high-risk groups and the management of screen-detected nodules. Based on a comprehensive balance between the benefits and potential harms of lung cancer screening, it emphasizes the critical importance of informed consent and shared decision-making. This revised guideline will be more aligned with China’s national conditions and enhance the standardization and applicability of LDCT-based lung cancer screening across the country.

肺癌是中国发病率和死亡率增长最快、对人群健康和生命威胁最大的恶性肿瘤。2022年，全国新发肺癌病例约106万，死亡病例超过73万，占全部恶性肿瘤发病和死亡的22.0%与28.5%。2000-2018年间，我国肺癌年龄标化发病率在男性和女性中均呈现显著上升趋势^[1]^。尽管近年来空气污染状况有所改善，但由于吸烟率持续偏高以及人口老龄化进程加快，预计未来几十年内我国肺癌负担仍将进一步加重。

肺癌的预后与临床分期密切相关，早期发现可显著提高治愈机会。国内以医院为基础的多中心研究^[2,3]^显示，对于I期肺癌患者，5年总体生存率可达82.3%，而晚期患者则显著下降，但我国诊断为I期肺癌的患者比例低于20%。2003-2005年至2012-2015年间，我国肺癌患者的5年生存率仅从16.1%提升至19.7%，改善有限^[4]^。主要原因仍在于早期诊断率较低，治疗时机延误，多数患者在确诊时已处于晚期，丧失了外科手术治疗机会。因此，推动肺癌的早期诊断和治疗，是提高肺癌5年生存率及改善患者预后的关键措施。

低剂量计算机断层扫描（low-dose computed tomography, LDCT）是目前唯一被证实可有效降低肺癌死亡率的筛查方法。美国国家肺癌筛查试验（National Lung Screening Trial, NLST）以及欧洲的荷兰-比利时肺癌筛查试验（Nederlands-Leuvens Longkanker Screenings Onderzoek, NELSON）等大型随机对照研究^[5,6]^均证实，LDCT筛查可有效识别早期肺癌，并降低20%-31%的肺癌相关死亡率。面对肺癌带来的沉重疾病负担，我国自2009年起陆续启动农村和城市肺癌筛查项目，各地亦逐步开展了LDCT筛查相关的公共卫生服务与研究^[7,8]^。随着筛查范围的扩大，更多早期肺癌患者被检出并得以接受手术治疗。近年来，尽管肺癌发病率仍在上升，其死亡率已出现明显下降；相应地，我国肺癌年龄标化5年相对生存率亦显著提高，在2019-2021年达到28.7%^[9]^。

随着LDCT筛查的临床价值得到充分验证，如何进一步提高筛查效率、降低其潜在风险，已成为当前肺癌防控领域的研究热点和核心发展方向^[10,11]^。为此，本文系统梳理了近两年国内外LDCT筛查的最新研究进展，以期为2025年版相关指南的修订提供科学依据。

## 1 参与本指南修订的人员结构及修订方法

本版指南的更新工作由中国肺癌早诊早治专家组与中国西部肺癌研究协作中心的专家联合完成。参与专家覆盖胸外科、肿瘤内科、影像学、病理学及流行病学等多个领域。农村肺癌早诊早治项目最初属于国家中央补助地方公共卫生项目，其专家组成员由国家卫生健康委员会批准指定。

本次指南修订主要参考2015、2018、2023年版LDCT肺癌筛查指南及近年来国外相关指南^[12-21]^。具体修订流程如下：首先在PubMed、Embase、Web of Science、万方数据、中国知网等数据库，检索2023年1月之后发表的肺癌筛查、早期诊断相关随机对照试验、系统评价与*meta*分析、指南及共识类文献；随后结合我国LDCT在城市与农村的肺癌筛查实践，系统评估LDCT筛查的获益与风险、适宜人群、筛查间隔及结节管理策略，并围绕上述核心内容对2023年版指南展开修订。

## 2 高危人群的选择

与2023年版肺癌LDCT筛查指南类似，近年来国内外多个肺癌筛查指南或共识均将吸烟外的其他风险因素也纳入了高危人群选择标准，但在筛查起始年龄、终止年龄、吸烟暴露强度（包年数）及戒烟时长等方面的推荐仍存在差异^[15,17-21]^。我国肺癌的年龄别发病率及死亡率在45岁之后显著增加，因此肺癌筛查与早诊早治方案（2024年版）^[21]^、早期肺癌诊断中国专家共识（2023年版）^[18]^和2024、2025版中华医学会肺癌临床诊疗指南^[19,20]^中，推荐肺癌筛查的起始年龄为40或45岁。然而，2014年全国肿瘤登记数据^[22,23]^显示，肺癌年龄别发病率逐渐上升，45-49岁肺癌年龄别发病率仅为全年龄组平均水平的50.0%左右，并且低年龄组发生过度诊断比例更高。因此，本次修订中推荐肺癌的开始筛查年龄仍为50岁。针对2023年版指南中“长期职业暴露”难以量化的问题，本次修订明确：一般职业暴露年限≥5年；若暴露强度高，则缩短至1年及以上即可视为符合筛查条件。

因此，在本次修订中，建议同时符合以下两项条件者参加肺癌筛查：（1）原则上年龄介于50-80岁。对于筛查终止年龄，应重视个体化风险评估，要根据受检者的整体健康状况、预期寿命及治疗耐受性进行个体化判别。若在年度筛查中发现因身体状况无法耐受可能的肺癌切除手术，或罹患严重威胁生命的疾病，则建议停止LDCT筛查；（2）具有下列条件之一：①吸烟史：吸烟≥20包年（每日吸烟包数×吸烟年数），或被动吸烟≥20年；如现已戒烟，戒烟时间≤5年者可继续筛查，超过5年者可建议停止；②职业致癌物暴露史：累计暴露于氡、砷、铍、铬及其化合物、石棉、氯甲醚、二氧化硅、焦炉逸散物或煤烟等明确肺癌相关职业致癌物≥5年；若暴露强度较高，≥1年亦可符合条件；③家族史合并吸烟暴露：一级、二级亲属患肺癌，同时吸烟≥15包年或者被动吸烟≥15年；④高发地区其他危险因素：对于某些肺癌高发地区，存在其他经证实的重要危险因素，亦可将其纳入高危人群的筛选条件。

此外，LDCT筛查的禁忌证与2018、2023年版指南保持一致，未作调整。

## 3 筛查间隔

当前，多数肺癌筛查指南推荐对高危人群采用LDCT进行年度性筛查。然而，年度筛查这一频率选择缺乏充分的生物学机制支持，且对于所有符合条件个体是否均需接受每年1次LDCT筛查，目前仍存争议^[24]^。鉴于筛查结果阴性者在后续随访中罹患肺癌的风险相对较低，建议对连续2年LDCT筛查均为阴性的个体暂停筛查2年；而对筛查结果阳性的个体，则仍应维持每年1次的筛查频率。

## 4 LDCT扫描参数

在应用X线电离辐射进行医学检查时，长期以来遵循“最低合理剂量（as low as reasonably achievable, ALARA）”原则，即将检查的辐射剂量尽可能地降低。该原则强调采用最低的放射剂量来获得合适的图像质量以用于准确诊断。临床实践已证实，剂量并非越低越好，剂量过低将不可避免地导致图像质量下降，造成误诊甚至无法用于诊断，而需要重新扫描。如果重复扫描，受检者的实际接受剂量则反而更高。显然，不能以牺牲诊断的准确性和医疗质量而去一味地追求低剂量，而是应该根据检查的目的，在剂量和图像质量二者间进行必要的权衡。影像学检查应从“低剂量”转向“right dose”：在准确记录剂量的基础上，为每位受检者选择最适扫描条件，兼顾ALARA、最安全操作（procedures as safe as reasonably achievable, ASARA）与最大获益（benefits as high as reasonably achievable, AHARA），以最低可实现的辐射换取最高质量的诊断与安全收益。

基于上述原则，扫描参数建议为：

### 4.1 扫描剂量

采用CT容积扫描技术，管电压采用110 kVp；管电流40 mAs，依据受检者体型，根据不同体重指数（body mass index, BMI）做一定范围内的调整。

对于体型较小的受检者（BMI≤30 kg/m^2^），建议扫描条件为100-120 kVp，管电流≤40 mAs，射线总剂量≤0.2 mSv；对于体型较大的受检者（BMI>30 kg/m^2^），建议扫描条件为120 kVp，管电流≤60 mAs，射线总剂量≤0.5 mSv。如果CT设备达不到0.2 mSv射线总剂量的标准，也可将射线总剂量在一定范围内做适当放宽调整，但仍保持在低剂量的标准范围内，即：对于体型较小的受检者（BMI≤30 kg/m^2^），扫描技术参数建议为管电压80-100 kVp，管电流≤40 mAs，射线总剂量0.5-1.0 mSv；对于体型较大的受检者（BMI>30 kg/m^2^），扫描技术参数建议为管电压100-120 kVp，管电流≤60 mAs，射线总剂量≤1.5 mSv。

### 4.2 扫描范围

从肺尖到肋膈角（包括全部肺），患者吸气末一次屏气完成扫描。

### 4.3 扫描后原始数据处理

行薄层重建，重建层厚为0.625 -1.250 mm。为方便进行计算机辅助检测（computer aided diagnosis, CAD）及容积分析，建议层间有20%-30%重叠。

### 4.4 薄层重建算法

建议采用软组织密度或肺算法，不建议采用高分辨率骨算法，因其对软件容积分析重复性影响较大。

### 4.5 肺结节的检测

建议将薄层图像行三维重建，采用最大密度投影（maximal intensity projection, MIP）重建，有助于结节的检出及结节形态的观察。推荐应用CAD软件结合人工阅片，以提高结节检出率。

### 4.6 图像后处理方法

对可疑肺癌的微小磨玻璃结节采用多平面重组（multiplanar reformation, MPR）、MIP、曲面图像重组（curved planar reformation, CPR）进行图像后处理，这能更有利于观察结节内部血管结构、周围边缘及移动血管的显示，以明确是否符合小肺癌的影像诊断。

## 5 结节管理

肺结节的恶性风险与其大小和生长速度均密切相关。目前，大多数筛查指南主要依据结节大小进行风险评估。近年来研究^[25]^显示，与测量最大直径相比，采用平均直径能够更准确地区分肺结节的恶性风险。此外，适当提高判定阳性结节的直径阈值，有助于显著降低CT筛查的假阳性率，减少不必要的医疗资源消耗^[26,27]^。基于上述研究进展，国际早期肺癌行动计划（International Early Lung Cancer Action Program, I-ELCAP）筛查方案、美国国立综合癌症网络（National Comprehensive Cancer Network, NCCN）肺癌筛查指南以及国内近年更新的相关指南均已推荐将平均直径作为评估结节大小的标准指标，并将基线筛查中实性结节或部分实性结节实性成分的阳性阈值提高至6 mm^[15,28]^。

本次修改也采纳了上述依据，具体结节管理方案如下。

### 5.1 阳性结节定义

对于LDCT筛查发现的不确定结节或非钙化性结节，根据结节性质及大小确定随访原则，并根据随访中结节的生长特性确定是否进行临床干预。

基线筛查阳性结节定义：实性结节平均直径或部分实性结节实性成分平均直径≥6 mm，或非实性结节直径≥8 mm，或发现气管或/及支气管可疑病变，或LDCT诊断为肺癌的肺部单发、多发结节或肺癌包块，定义为基线筛查阳性，应当进入临床治疗程序。

年度筛查：发现新的非钙化性结节或气道病变，或发现原有的结节增大或实性成分增加，则定义为年度筛查阳性。

### 5.2 结节管理和进一步诊断

#### 5.2.1 基线筛查

CT检查阴性者（未检出不确定结节或非钙化性结节；实性结节平均直径或部分实性结节实性成分平均直径<6 mm；或非实性结节平均直径<8 mm），12个月后按计划进入下一年度复查。

平均直径≥6 mm且<15 mm的实性结节，或实性成分平均直径≥6 mm且<15 mm的部分实性结节，筛查3-6个月后进行复查。平均直径≥8 mm且<15 mm非实性结节，筛查6个月后进行复查。如果结节增大，由临床多学科诊疗（multi-disciplinary treatment, MDT）团队会诊，决定是否进入临床MDT治疗；如结节无变化或缩小，进入下一年度复查。

对于平均直径≥15 mm的结节，有两种方案：（1）由临床MDT团队会诊，决定是否进入临床MDT治疗；（2）抗炎治疗2-3周，休息1个月后复查。如果病灶完全吸收，进入下一年度复查；如果结节无变化，由临床MDT团队会诊，决定是否进入临床MDT治疗；如果结节部分吸收，6个月后进行CT复查，结节增大者，由临床MDT团队会诊决定是否进入临床MDT治疗；结节缩小或无变化者，进入下一年度复查。

LDCT诊断为肺癌的肺部单发、多发结节或肺癌包块，应当进入临床治疗MDT程序。LDCT筛查如发现气管或/及支气管可疑病变，应进行临床MDT干预，并进行纤维支气管镜检查，并在必要时进一步随诊（图1）。

**图 1:**
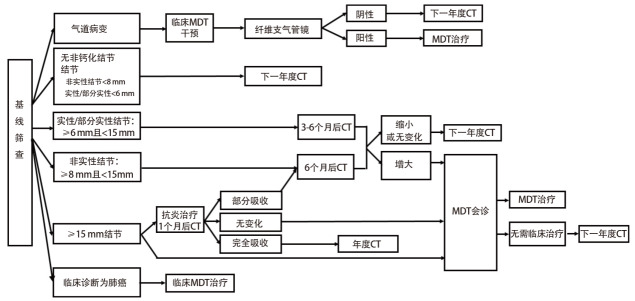
基线筛查流程及结节管理

#### 5.2.2 年度筛查结节管理

对于年度CT复查发现新的非钙化性结节，6个月后复查，如果复查结节增大，由临床MDT团队决定是否进入MDT治疗；如果未增大，进入下一年度复查（图2）。

**图 2:**
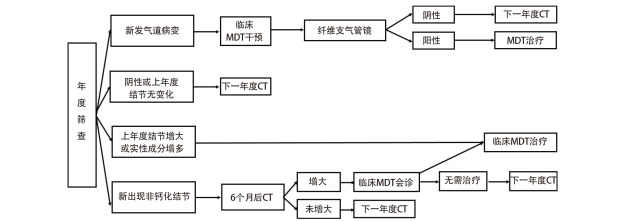
年度筛查流程及结节管理

年度复查发现原有的肺部结节明显增大或实性成分明显增多时，应进入全程管理的临床MDT治疗程序。

年度筛查中发现的气管或/及支气管可疑病变，处理同基线筛查。

### 5.3 临床干预

包括以下几种情况：（1）LDCT检查发现气道病变者：应行纤维支气管镜检查。纤维支气管镜检查阳性且适合于外科手术治疗者，应行外科手术为主的MDT综合治疗。纤维支气管镜检查阴性者，则进入下一年度复查。（2）LDCT诊断为肺癌或高度疑似肺癌者：①LDCT筛查高度怀疑为肺癌的阳性结节者，应当由高年资的胸外科、肿瘤内科、呼吸科和影像科医师进行MDT会诊，决定是否需要进行临床治疗以及采取何种方法进行治疗。对于适合于外科手术治疗者，一定首选外科治疗。②LDCT诊断为肺癌的单发、多发结节或肺癌包块，应进入临床治疗程序，经临床检查适合外科手术治疗者，行外科手术为主的MDT治疗。（3）LDCT诊断为肺癌或高度怀疑为肺癌的肺部单发、多发结节或肺部包块：由于肿瘤原因、患者心肺功能异常不能耐受外科手术治疗，或者患者本人不愿意接受外科手术治疗者，为明确病变性质行纤维支气管镜细胞学刷检、活检，或者行跨支气管壁纵隔淋巴结穿刺活检等病理学或细胞学检查；对于纤维支气管镜检查不能获得细胞学或者病理学诊断者，可以行经皮肺穿刺活检标本送病理检查。上述方法获得的肿瘤组织标本，除了行 病理学或者细胞学检查外，应行下一代测序（next-generation sequencing, NGS）检查，以便确定患者是否适合分子靶向治疗。通过上述方法获得细胞学、病理学检查明确肺癌诊断及驱动基因检测结果后，应当组织MDT团队讨论，给予肺癌治疗全程管理的“个体化”多学科综合治疗。

## 6 知情同意与共同决策

高危人群的界定、筛查间隔的设置与阳性结节的管理是LDCT肺癌筛查中的三个关键环节。在2023年版中，建议高危个体在参与筛查前与医疗专业人员充分沟通LDCT筛查的潜在获益与风险，共同做出决策。考虑到若延长低危个体的筛查间隔，可能增加假阴性风险；同时，部分肺结节（尤其是非实性结节）存在过度诊断与过度治疗的可能，本次修订进一步强调，应在上述三个环节中均贯彻共同决策理念，由高危个体与医务人员就各方面可能的获益与风险进行充分讨论。

## 7 筛查与戒烟结合

对于目前吸烟者，应明确强调戒烟是降低肺癌风险最根本和有效的措施。将戒烟与LDCT肺癌筛查相结合，是最大限度降低肺癌死亡风险的最佳策略。参加肺癌筛查为吸烟者提供了接受戒烟教育的机会。建议将戒烟咨询和宣传整合到肺癌筛查的全流程中，为筛查者提供系统、持续的戒烟支持。

## 8 讨论

自本指南2023年版发布以来，国内外开展了大量的优化LDCT筛查策略的研究。基于近年来国内外LDCT肺癌筛查进展，结合我国肺癌流行病学特征，本版本对以下方面进行了修订：（1）高危人群定义中明确了职业暴露年限，另外，如果在年度性筛查中身体状况不能耐受可能的肺癌切除手术或罹患了可严重影响生命的疾病的患者群体，则建议停止LDCT筛查；（2）更新了结节大小的测量方法，将平均直径作为结节大小的衡量指标；（3）结节管理中，将基线筛查中实性结节或部分实性结节实性成分的平均直径阳性阈值提高至6 mm，并将实性结节或部分实性的阳性结节复查时间由6个月调整为3-6个月；（4）建议将知情同意和共同决策贯穿于高危人群选择、筛查间隔和结节管理环节。本次修订进一步明确和优化了高危人群选择及结节管理，并在对肺癌筛查获益和危害的充分权衡基础上，强调了知情同意和共同决策的重要性。

肺癌筛查的收益随着筛查人群肺癌发生风险的增加而增加。因此，目前国内外肺癌筛查指南或共识均建议在高风险人群中进行肺癌筛查。2023年美国癌症协会肺癌筛查指南和最新的NCCN肺癌筛查指南认为吸烟人群戒烟15年后其肺癌发病率仍显著高于非吸烟者，在界定高危人群时取消了对戒烟者戒烟时间的限制^[15,16]^。然而，我国吸烟者发生肺癌的相对危险度显著低于西方人群，因此我们仍保留了戒烟年限不超过5年的限制。此外，尽管没有调整筛查起止年龄，但本次指南明确了如果患者身体状况不能耐受可能的肺癌切除手术或罹患了可严重影响生命的疾病，则建议终止LDCT筛查。

近年来，越来越多的肺癌筛查指南的高危人群纳入标准考虑了年龄和吸烟之外其他危险因素，如职业暴露。但目前指南中对职业暴露史的判定存在差异，多数指南仅定义为“长期职业暴露”，在实践中难以统一标准。国家肺癌筛查指南2021年版中定义职业暴露年限为至少1年^[17]^。然而，除短期内的高强度暴露外，职业暴露年限超过5年则肺癌的风险会显著增加^[29]^。因此本次修订中明确了职业暴露年限为至少5年。

结节的准确测量和结节管理是LDCT筛查的核心环节。平均直径作为一种更科学、准确、稳定的评估方式，已逐步取代最大直径，被建议用于肺癌筛查中结节大小的测量^[30,31]^。本次修订也将结节的测量方式由最大直径修改为平均直径，对于部分实性结节，改为了实性部分平均直径的测量。在结节管理中，本次修订借鉴了NCCN及国内相关筛查指南，将基线筛查中实性结节的平均直径和部分实性结节实性部分平均直径的阳性阈值从5 mm提高至6 mm。一项基于NLST的数据^[26]^比较了不同结节分类标准的肺癌漏诊和假阳性情况，结果显示，与4 mm的结节分类标准相比，6 mm结节分类标准会导致1.5%的肺癌诊断延迟，但会减少36.8%的假阳性。I-ELCAP研究结果也与此类似^[30]^。因此，将基线筛查中实性结节的平均直径和部分实性结节实性部分平均直径阳性阈值提高至6 mm有助于在不影响获益的情况下降低LDCT筛查的假阳性。

惰性肺癌的过度诊断是LDCT筛查的一种重要潜在危害，可导致医疗费用、焦虑心理和癌症诊断、治疗相关并发症的额外增加^[32,33]^。目前肺癌筛查研究中过度诊断率的报告结果并不一致。NLST试验^[34]^表明，肺癌筛查中过度诊断主要来自于早期腺癌。其通常由亚实性结节发展而来，并且在老年女性和亚洲人群中更为常见。随着我国肺癌筛查指南中将被动吸烟等其他危险因素纳入高危标准，我国女性肺癌发病率近年来显著上升。我国的研究发现在女性肺癌中早期肺癌发病率显著增高，而晚期肺癌发病率并无下降，提示存在过度诊断^[23,35]^。因此，今后我国LDCT筛查的过度诊断率可能会高于西方国家水平。对非实性肺结节进行保守性的主动监测，有助于避免大部分惰性或生长缓慢的肺癌被不必要的手术切除，而不影响筛查的效果^[36]^。我国一项10年随访研究^[37]^表明，对于非实性结节（纯磨玻璃结节），无论随访期间是否增大，持续CT监测与立即手术在10年总生存率上无显著差异，因此可安全随访至出现实性成分再考虑手术。另一项前瞻性多中心临床研究^[38]^也表明，对特定类型的肺部多发性磨玻璃结节患者，在“外科治愈窗口期”内实施主动监测策略安全可靠。在本次修订中，我们建议对于非实性结节，尤其是年龄较轻的女性，需要与其进行讨论并共同决策，以避免过度外科干预。

国外研究基于年龄、性别、种族、教育水平、BMI、家族史及吸烟史等因素，建立了多种肺癌风险预测模型，但这些模型主要基于吸烟人群构建。为应对中外肺癌流行病学与临床特征差异，近年来我国也逐步开发了同时涵盖吸烟与非吸烟个体的肺癌风险预测模型^[39-41]^。例如，国家癌症中心基于国家肺癌筛查项目，建立了包含吸烟者与非吸烟者的肺癌3年风险预测模型^[39]^。另一项研究^[40]^则利用中国慢性病前瞻性研究队列，构建并验证了一个适用于各年龄段、吸烟与非吸烟人群的肺癌风险预测工具。目前，NCCN肺癌筛查指南推荐使用Tammemägi肺癌风险计算器，以帮助量化人群的肺癌风险^[15]^。然而，结合我国实际情况，本次指南修订中认为当前的肺癌风险预测模型仍需更多前瞻性研究验证，以进一步积累证据支持其用于组织性人群筛查。

在我国LDCT筛查范围持续扩大的背景下，为应对我国肺癌流行病学特征复杂、地域差异显著的现实挑战，亟需向“精准化筛查”转型。当前，分子标志物、影像组学、人工智能等新兴技术在肺癌筛查领域的研究与应用不断深入，有助于从高危人群选择、筛查间隔及阳性结节鉴别分流等方面优化肺癌筛查策略，从而使肺癌筛查走向“个体化”和“精准化”^[42-44]^。

## 9 结语

本研究基于近年来国内外肺癌筛查的最新进展与我国肺癌流行趋势，兼顾组织性筛查的规范性与普适性需求，对2023年版LDCT筛查指南进行了修订。修订重点聚焦于高危人群标准的细化，并系统优化了结节管理与共同决策这两个关键环节，旨在为我国肺癌筛查实践提供更科学、规范的参考。

**指南编写组成员**

**执笔专家（排名不分先后）：**周清华（四川大学华西医院肺癌中心/肺癌研究所），范亚光（天津市肺癌研究所，天津医科大学总医院），乔友林（中国医学科学院/北京协和医学院群医学及公共卫生学院），张国桢（复旦大学附属华东医院）

**专家组成员（排名不分先后）：**周清华（四川大学华西医院肺癌中心/肺癌研究所），范亚光（天津市肺癌研究所，天津医科大学总医院），乔友林（中国医学科学院/北京协和医学院群医学及公共卫生学院），张国桢（复旦大学附属华东医院），黄云超（云南省肿瘤医院，昆明医科大学第三附属医院，云南省癌症中心），王颖（天津医科大学总医院），李铭（复旦大学附属华东医院），林冬梅（北京大学肿瘤医院），王洁（中国医学科学院肿瘤医院），孙燕（中国医学科学院肿瘤医院），王新允（天津医科大学总医院），郑向鹏（复旦大学附属华东医院），李为民（四川大学华西医院），车国卫（四川大学华西医院），许峰（四川大学华西医院），李潞（四川大学华西医院），朱大兴（四川大学华西医院），陈良安（中国人民解放军总医院），刘洁薇（四川大学华西医院），胡成平（中南大学湘雅医院），王艇（四川大学华西医院），王琪（大连医科大学第二医院），阴丽媛（四川大学华西医院），刘刚（吉林省肿瘤医院），蒋莉莉（四川大学华西医院），张蕾（中国医学科学院肿瘤医院），刘曙正（河南省肿瘤医院），王贵齐（中国医学科学院肿瘤医院），王强（吉林省肿瘤医院），吴宁（中国医学科学院肿瘤医院），王永生（四川大学华西医院），邓汉宇（四川大学华西医院），梅婷（四川大学华西医院），黄开利（四川大学华西医院），付军科（西安交通大学第一附属医院），唐小军（四川大学华西医院）
